# The combined use of in vivo confocal microscopy and optical coherence tomography in monitoring corneal infections

**DOI:** 10.1038/s41433-026-04599-7

**Published:** 2026-06-09

**Authors:** Farida Omar ElZawahry, Sarah Donatelli, Mario Nubile, Prity Sahay, Dalia G. Said, Harminder S. Dua

**Affiliations:** 1 Division of Clinical Neuroscience, Department of Ophthalmology, University of Nottingham, Nottingham, UK; 2 National Institute of Laser enhanced Sciences, Cairo University, Cairo, Egypt; 3 University of Degli Studi G.D’Annuzio, Chieti Pescara, Italy

**Keywords:** Corneal diseases, Infection

## Abstract

**Background/Aims:**

Infectious keratitis (IK) is a major cause of blindness worldwide, requiring prompt diagnosis and management. This study evaluates the diagnostic and evaluative roles of in vivo confocal microscopy (IVCM) and anterior segment optical coherence tomography (AS-OCT) in acute IK through correlating the characteristic imaging features of both modalities.

**Materials and methods:**

In this prospective study, 35 eyes with severe IK of bacterial, fungal, Acanthamoeba, or mixed origin were examined using slit lamp biomicroscopy, microbiological culture, AS-OCT, and IVCM during the acute phase. Imaging parameters including tissue integrity, cellular infiltration, nerve presence, vascularisation, and microbial features were analysed. Quantitative cell density measurements and structural assessments were performed, with statistical comparisons between modalities and pathogen groups.

**Results:**

IVCM effectively visualised cellular details, including keratocytes, inflammatory cells, nerve fibres, and specific organisms, with high sensitivity for detecting fungal filaments and Acanthamoeba cysts. AS-OCT provided high-resolution cross-sectional images, accurately measuring infiltrate depth, corneal thickness, and neovascularisation. IVCM was superior in identifying microbial elements, whereas AS-OCT excelled in assessing tissue architecture and oedema. Significant differences in cell densities were observed among pathogens, notably lower keratocyte density in Acanthamoeba keratitis. Both modalities were complementary, enhancing disease characterisation and monitoring.

**Conclusions:**

IVCM and AS-OCT are valuable, non-invasive tools that provide critical insights into IK. Their combined application improves early diagnosis, detailed evaluation, and treatment monitoring. Integration with artificial intelligence holds promise for further advances in precise, rapid diagnosis and personalised management of infectious keratitis.

## Introduction

Infectious keratitis (IK) is among the top five leading causes of blindness globally. Early diagnosis is needed to guide appropriate therapy to avoid unfavourable complications such as vision impairment and blindness [1]. İt has been recognised as a “silent epidemic” in the developing world [2]. It is a disease characterised by acute ocular pain, decreased vision, corneal ulceration, and/or stromal infiltrates. Keratitis is considered as an acute ophthalmologic condition and requires urgent treatment [1, 3].

Slit lamp microscopy and culture of corneal scrapes are key to diagnosing IK [4, 5]. Alternative diagnostic tools include imaging, such as optical coherence tomography (OCT) and in vivo confocal microscopy (IVCM), which are two very useful imaging modalities in examination of the cornea. OCT provides non-contact, cross sectional images with a wide angle of view, under high magnification, simulating histology but in fact is dependent on the reflectivity (refractive index) of tissues, which gives it the ability to accurately determine the depth and extent of the corneal ulceration, infiltrates and haze, enabling characterising the severity and progression of the infection [6]. However, OCT is not a preferred choice in the diagnostic tool package for infectious keratitis as imaging through scars and dense infiltrates is poor on account of the back shadowing, which masks details [4, 7, 8].

IVCM provides en-face images with a restricted field of view but with a resolution range of 1.5–4 μm allowing examination at a cellular level. The different layers of the epithelium, sub-basal nerve plexus, Bowman’s zone, stroma with keratocytes and nerves, the Descemet’s membrane and endothelial cells can be clearly visualised in health and disease. Besides providing detailed morphology of the above structures it also enables visualisation of invading organisms such as Acanthamoeba and fungi with overall sensitivities of 66–74% and 80–100% and specificity of 78–100% and 84–100%, respectively [9–14].

Software associated with both OCT and IVCM allow measurement of dimensions of objects/area (s) of interest. OCT is more accurate in measurement of depth while IVCM enables quantification of cell size, shape and numbers. Interpretation skills require experience for both [14–16].

Recently, deep learning (DL) models have been shown to be promising aids for the diagnosis of IK via image recognition [17, 18]. Most of the studies that have developed DL models to diagnose the different types of IK have utilised slit lamp photographs. Some studies have used extremely efficient single convolutional neural network algorithms to train their models, and others used ensemble approaches with variable results [10]. Limitations of DL models include the need for large image datasets to train the models, the difficulty in finding special features of the different types of IK. The imbalance of training models, the lack of image protocols and misclassification bias, need to be overcome to apply these models into real-world settings [19–21].

We undertook consecutive Anterior segment optical coherence tomography (AS-OCT) and IVCM examination of cases of IK in the acute stage and correlated the scans in all phases to establish features that can help in the diagnosis, monitoring, and evaluation of response to treatment, in these cases.

## Materials and methods

### Study design and participants

This single centre observational study was conducted at Queen’s Medical Centre, Nottingham. The study adhered to the Declaration of Helsinki and its later amendments, and informed consent was obtained from participants. Ethical committee approval ID: 152709.

IVCM and AS-OCT images of 35 consecutive patients (35 eyes) who presented with severe IK (sight threatening, requiring hospital admission for intensive medication) between January 2023 and December 2024 were evaluated. Patients included Acanthamoeba, fungal, bacterial and mixed keratitis. Types of infections and their distribution are reported in Table 1.

**Table 1:** Shows the types and distribution of infectious organisms in our study population.

Type of infection	Number of eyes (*n* = 35)	Percentage of total (%)
Bacterial	10	28.58
Fungal	6	17.14
Protozoan (Acanthamoeba spp.)	6	17.14
Combined bacterial and fungal*	2	5.71
Combined bacterial and protozoan*	2	5.71
Unidentified pathological organism	8	25.72

Total = 35 eyes.

*Only two organisms were detected (bacterial + fungal = 2) and (bacterial + Acanthamoeba =2).

All patients were treated in accordance with our standard protocols for bacterial, fungal, parasitic (Acanthamoeba) and mixed infections. AS-OCT, IVCM and Slit lamp photography were performed on initial presentation. We were able to obtain simultaneous slit-lamp, IVCM, and AS-OCT images of the lesions, allowing qualitative multimodal comparison at the same clinical time point. Only one patient underwent previous corneal surgery and have an infected DALK graft.

### Inclusion and exclusion criteria

IVCM and AS-OCT images of 35 consecutive patients (35 eyes) who presented with severe IK (sight threatening, requiring hospital admission for intensive medication) between January 2023 and December 2024 were evaluated. Patients with sufficient clinical/ microbiological findings to support the diagnosis of Acanthamoeba, fungal, bacterial and mixed keratitis were included. Patients who were unable to undergo imaging, producing poor-quality scans, and cases with non-infectious keratitis and those lacking adequate clinical or microbiological documentation were excluded from the study.

### Clinical and microbiological assessment

For all participants, corneal slit lamp biomicroscopy was undertaken by an experienced corneal specialist, microbiological culture and scrapings were obtained prior to initial scanning and AS-OCT scans and IVCM scans, using the anterior segment module of the HRT II (Heidelberg retina tomogram, Heidelberg Engineering GmbH, Heidelberg, Germany) (HEYEX I) software was used in scotopic conditions. Forty-two horizontal volume scans of 278 microns intervals, and width of 20°x10° (11.1 mm×5.6 mm) to cover the area of infection, were captured. The anterior segment module of the Heidelberg Retina Tomography 3 ((HRTIII) Rostock cornea module, Heidelberg Engineering GmbH, Heidelberg, Germany) confocal microscopy, were obtained and evaluated concurrently.

### Imaging protocol

AS-OCT, IVCM and Slit lamp photography were performed on initial presentation. (The acute stage of infection).

IVCM was performed by using a diode-laser 670 nm wavelength. The IVCM analysis included the evaluation of the corneal morphology, and determination of cell density (Keratocyte and dendritic cells).

Cell density was counted in the anterior stroma (at depth of 50–150 micron from corneal surface) and posterior stroma (at depth of 300–400 micron from corneal surface). In cases of corneal stromal ulceration IVCM analysis was focused at the level of the bottom of the stromal defect whenever possible. At least 30 images of both anterior and posterior stroma were acquired. Cell density was considered as an average of four measurements in different representative frames. Endothelial cell count was determined as an average of four different Images, where applicable.

Cell density was calculated using the analysis software of the instrument, by averaging the numbers of cells from the selected images in each position, counted manually within a region of interest of standard dimensions (250 × 250 μm). Cell densities were given as cells per square millimetre.

Confocal microscopic scanning was performed in five different corneal areas independently from the location of the corneal lesion. At least four confocal images at the level of the basal lamina were obtained in each of the following corneal zones: central, superior, inferior, nasal, and temporal quadrant.

### Image analysis and outcomes

The following categorical parameters were evaluated for both IVCM and their corresponding AS-OCT scans: epithelial defects, epithelial thickness, subbasal nerve plexus, the depth of the infiltrate and the corneal thickness, presence of neovascularisation (recognised on AS-OCT as the “barcode sign” [22, 23], cellularity and cell density (dendritic cells, hyphae, in cases of fungal keratitis, and Acanthamoeba cysts and/or trophozoites in Acanthamoeba keratitis, inflammatory cells and activated keratocytes), stromal melting, oedema, endothelium, keratic precipitates (KPs), hypopyon, and endothelial plaques.

The selected images were the best focused with the whole image in the same layer and good contrast. Images were de-identified before analysis to avoid observer bias.

One masked observer analysed all IVCM images similar to a previous report [21].

### Statistical analysis

Statistical analysis was performed using GraphPad Prism 8.2.1 (GraphPad Software, Inc., San Diego, CA, USA). Descriptive statistics were computed for all continuous variables. Fisher’s exact test and odds ratio (OR) were used for categorical variables. Odds ratios were calculated as the odds of detection by IVCM relative to AS-OCT. Data was analysed by the 2-way ANOVA test with Tukey’s multiple comparisons test was used to compare stromal cell density according to two factors: organism group (bacterial, fungal, Acanthamoeba) and stromal depth (superficial, deep). Where cell density could not be quantified because of a hyperreflective background, those images/cases were excluded from quantitative cell density analysis but retained for qualitative descriptive analysis, test with a *p* value < 0.05 was considered to indicate statistical significance.

## Results

### Findings on IVCM and AS-OCT

Overall, 35 eyes from 35 patients were included in the study (17 males and 18 females, mean age 47, range 16–78). The diagnosis of infectious keratitis was mainly clinical, however 20 of 35 patients were culture positive. Types of infections based on microbial diagnosis are reported in Table 1. One patient/ eye underwent previous surgery *n* = 2.86%.

Compared to IVCM, AS-OCT was significantly more likely to detect the depth of the infiltrate (OR 0.16, *p* = 0.0007), presence of hypopyon (OR 0.0, *p* = 0.0029), and presence of endothelial plaques (OR 0.06, *p* = 0.0029). Although corneal thickness was more frequently established using AS-OCT, this was not significant (OR 0.35, *p* = 0.10).

IVCM was more likely to detect nerve density (OR infinity, *p* < 0.0001) and microbial cells (OR infinity, *p* < 0.0001). Neovascularisation (OR 1.13) and melting (OR 1.84) were more frequently detected on IVCM but the findings were not statistically significant (*p* > 0.99 and *p* = 0.32, respectively).

No significant differences were detected for epithelial alterations (OR 0.86, *p* > 0.99).

### Cell density evaluation on IVCM

We further compared superficial and deep stromal cell density among the different organism groups. The area of the ulcer showed the absence of corneal epithelium, basal lamina, and Bowman’s membrane. At the bottom of the corneal ulcer, an increased reflectivity of the stromal tissue was observed, suggesting the presence of issue necrosis. No nerves were detectable in most cases; the inter and intra cell density was measured in the superficial and deep stroma in each organism the compared between the superficial stroma and the deep stroma of the organisms respectively. Cell density could not be quantified due to hyperreflective background in six cases (*n* = 17.1%). s cases keratocytes and/or microbiological specific cells were detectable at the area of the lesion, while in other they were uncountable due to hyperreflective background. Quantitative analysis showed that in fungal keratitis (FK), the mean cell density in the superficial stroma was 371.1 ± 62.4 cells/mm², compared to 277.5 ± 46.5 cells/mm² in the deep stroma, demonstrating a statistically significant difference (*p* < 0.0001).

In Acanthamoeba keratitis (AK), superficial stromal cell density was 252.4 ± 63.9 cells/mm² while deep stromal density was higher at 316.9 ± 77.0 cells/mm², also showing a statistically significant difference (*p* = 0.0011).

In contrast, in bacterial keratitis (BK), superficial stromal cell density (204.5 ± 28.8 cells/mm², and deep stromal density (170.3 ± 22.6 cells/mm², did not differ significantly (*p* = 0.1192).

### Cell density difference in superficial stroma

Between-organism comparisons revealed that in the superficial stroma, FK (371.1 ± 62.4 cells/mm²) demonstrated significantly higher cell density compared to both BK (204.5 ± 28.8 cells/mm²) and AK (252.4 ± 63.9 cells/mm²) (*p* < 0.0001 for both comparisons).

### Cell density difference in deep stroma

In the deep stroma, FK (277.5 ± 46.5 cells/mm²) and AK (316.9 ± 77.0 cells/mm²) both showed significantly higher cell densities compared to BK (170.3 ± 22.6 cells/mm²) (*p* < 0.0001).

No statistically significant difference in deep stromal cell density was observed between AK and BK (*p* = 0.7165), nor between AK and FK (*p* = 0.029), despite a trend toward higher values in AK.

## Discussion

History and clinical examination with the slitlamp supported by microbiological examination including culture are the mainstay in the diagnosis of IK. The latter is affected by previous medications, quality of sample and the generally poor percentage of positive results. In our cohort, 20 of 35 cases (57.1%) were culture positive, which is consistent with previously reported culture positivity rates ranging from 33 to 66% in infectious keratitis with reports ranging from 33 to 66% with an average of less than 50% even in well developed facilities [3–5].

Atypical and overlapping clinical features, and mixed infections further compound the diagnostic conundrum. Adjunctive assessments with high resolution AS-OCT and IVCM are increasingly being used to improve and expedite accuracy in diagnosis [9, 10].

AS-OCT provides cross sectional images of the cornea, delineating tissues of different refractive indices thus providing a semblance of histological images and has proven to be useful in numerous corneal pathologies including IK [8, 22–24]. The cross-sectional images parallel the slit beam images of the slit lamp but at greater magnification. It is easy to perform, can be carried out by a trained technician; it has a non-contact approach, which makes it patient friendly; it takes very little time to perform scans that cover the entire cornea, hence can be done and evaluated as part of the patients’ clinic visit. It provides objective measurements of dimensions (height, width, and depth) and density of tissues. Dense lesions like scars, infiltrates and blood vessels, produce back shadows which obscure visualisation of structures covered by the shadow however, the diminishing of the shadows points to the resolution of the lesion and can be used as an objective measure [25].

IVCM on the other hand provides en-face images, at a cellular and sub-cellular level, of tissue structures through the thickness of the cornea [8, 10, 11, 14, 26, 27]. It has been considered an ‘in vivo corneal biopsy’, providing strong clues to histological diagnosis and often correlating with microbiological findings, particularly in suspected fungal or Acanthamoeba keratitis when culture results are negative. [28]. Different devices can be used in both non-contact and minimal contact manner. The latter requires use of topical anaesthesia and patient cooperation, which can be difficult in an actively infected and inflamed eye. It takes longer to perform, and due to the limited area covered, generate a substantial amount of images, which take time to study. It thus requires a trained technician to perform the scan and a trained doctor to interpret the images. Additionally, both imaging modalities show the corneal tissue from two different views. The IVCM used in this study HRT-RCM system is able to produce high quality images, with a lateral resolution of 1 μm, axial resolution of 7.6 μm and ×400 magnification while the AS-OCT produces high resolution images, but has a lateral resolution of 14 µm resolution. Which has an axial resolution of 3.9 μm.

In this study, we were able to perform and correlate slitlamp, IVCM and AS-OCT image of the lesion and cornea, at the same time points, which sets it apart from other studies on IK. All patients studied had severe IK requiring hospitalisation for intensive medication. This might be a limitation and a similar study on smaller, less severe IK is contemplated.

Most features of IK seen on slit lamp biomicroscopy were also noted in the AS-OCT scans but at higher magnification. IVCM on the other hand was able to show additional findings, such as microbial cells which were discernible in the majority of cases despite the density of the infiltrates and gross oedema. However, in cases with severe tissue necrosis a homogenous hypereflective background was noted which had a masking effect. Importantly, subepithelial and stromal corneal nerves were detected only on IVCM.

IVCM was able to detect presence/absence of normal keratocyte-like morphology of stellate interconnected cells, dendritiform cells (DFCs), inflammatory cells in a honeycomb distribution, and features of some organisms. Chidambaram et al. successfully used IVCM to determine cellular and structural features of fungal (FK), Acanthamoeba (AK), and bacterial keratitis (BK) [29]. Similarly, Sharma et al. used AS-OCT to monitor 50 patients with fungal keratitis at different time points and quantified different parameters to monitor the activity in cases with deep stromal involvement and endothelial plaques [26].

Al Aqaba et al. studied the corneal nerves using IVCM in depth, and reported that structural and functional nerve anomalies can be induced by corneal pathology, such as IK and conversely, nerve pathology can drive inflammation and corneal pathology, which can account for neurotrophic persistent epithelial defects post healing of IK [30]. Several studies have reported on corneal nerve involvement in corneal pathology [26, 30, 31]. Different changes occur in the corneal stroma during IK. We were able to quantify the dendritic and inflammatory cell density using IVCM. Indirect quantification of cell infiltration by densitometry studies on Pentacam Scheimpflug images in IK was carried out by Otri et al. showing that changes in densitometry is an important aspect of IK both in the active and resolving stages [32]. Early localised and diffuse necrotic stromal cystic spaces in fungal keratitis cases have been reported [32].

The clinical findings as visualised by AS-OCT and IVCM are reported in Table 2. AS-OCT and IVCM were complementary to each other. Though several parameters were seen by both devices, there were some better visualised by one, compared to the other. The most significant of these was the inability of AS-OCT to demonstrate corneal nerves due to the similar refractive indices of the nerves and stroma. IVCM on the other had provided clear images of the sub-basal plexus and stromal nerves. Several studies have reported on the corneal nerve morphology and morphometry in corneal health and disease [33]. In our study, in the ulcerated area, we noted nerves to the edge of the ulcer but not within, either due to masking with the infiltrate or destruction by the disease process.

**Table 2:** Shows the parameters assessed by both AS-OCT and IVCM.

Parameter	ASOCT	IVCM	Comments
Epithelial defect	+	+	Seen on both devices
Corneal nerves		+*	Not possible through ASOCT due to the homogenisty
Depth of the infiltrate	+*		of nerves with the stromal tissue reflectivity
Hyperreflectivity	+	+	Visible on both devices but better quantified on ASOCT
Inflammatory cells		+	
Cell density		+*	
Corneal vessels	+	+	Blood vessel and circulating blood seen on IVCM
Melting/ tissue necrosis		+	Seen as lacunae on IVCM
DM/ endothelial undulation	+*		Better seen on ASOCT
Hypopyon /endothelial plaque	+*		

^*^Statistical significant difference in detecting the specific finding between the two devices.

**Fig. 1:**
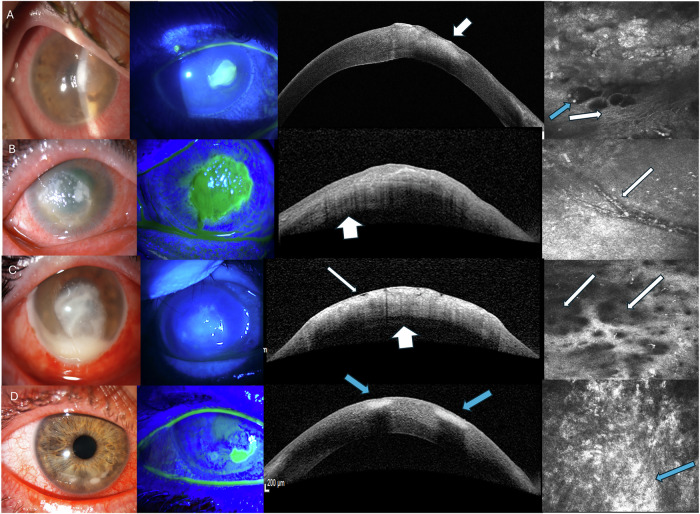
Multimodal imaging features of Infectious Keratitis: Slit-Lamp, Anterior Segment OCT, and In-Vivo Confocal Microscopy correlation across different etiologies. **A** Case of culture positive acanthamoeba keratitis. Slit lamp examination shows an infiltrate with an evident epithelial defect with positive fluorescein staining. Corresponding anterior segment optical coherence tomogram (AS-OCT) of the cornea at the acute stage showing an epithelial defect with a superficial dense hyper-reflective foci of infiltration (white arrow). In vivo confocal microscopy (IVCM) showing alterations of epithelial cells morphology with epithelial loss (white arrow), and evident anterior stromal melting/necrosis with tissue resorption (vacuoles) with some acanthamoeba cysts. (Blue arrow). **B** Images of a confirmed case of mixed acanthamoeba/ bacterial (pseudomonas) keratitis. Slit lamp examination showing a ring ulcer and paracentral infiltrates, and extensive corneal vascularisation and irregular edges with positive fluorescein staining. Corresponding anterior segment optical coherence tomogram (AS-OCT) of the cornea at the acute stage, with mid-stromal vascularisation distinctly showing “The barcode sign” produced by back-shadows of active corneal vessels (white arrow) In vivo confocal microscopy (IVCM) showing an active intrastromal blood vessel with visible circulating blood cells (white arrow) in a hypereflective stroma denoting the density of the infiltrate. Note the hyperefelctive acanthamaeba cysts seen superficially to the blood vessel. **C** Images of a confirmed case of fungal keratitis. Slit lamp examination showing a dense central infiltrate with feathery edges, corneal melting, and hypopyon. Corresponding anterior segment optical coherence tomogram (AS-OCT) of the cornea, showing areas of stromal necrosis (white arrows) which are visible despite the shadows cast by the infiltrate. In vivo confocal microscopy (IVCM) showing an irregular honeycomb like appearance (white arrows) caused by the formation of multiple, cystic spaces within the corneal stroma denoting tissue melting. This can result from the enzymatic degradation and breakdown of stromal lamellae by infectious agents leading to localised tissue destruction. (Suggesting an aggressive or advanced stage of infectious keratitis). **D**. Images of a confirmed case of filamentous fungal keratitis. Slit lamp examination showing paracentral infiltrates with positive fluorescein staining. Corresponding anterior segment optical coherence tomogram (AS-OCT) of the cornea at the acute stage, showing two dense, hyperreflective superficial stromal infiltrates casting backshadows (white arrows) In vivo confocal microscopy (IVCM) of the area of the infiltrate showing a hypereflective extracellular matrix corresponding to the density of the infiltrate. (Blue arrow).

Corneal vessels walls were visible directly with blood cells in the lumen, on IVCM scans. In contrast on AS-OCT the location of the vessel on cross section was indicated by a dense shadow posterior to it, extending through the rest of the thickness of the cornea, giving the classic ‘barcode sign’ which has been shown to be related to the blood in the vessel lumen as the shadowing effects decreases as the new vessels regress [22]. Corneal oedema was better seen with AS-OCT both in terms of corneal thickness measurement and the ‘saw tooth’ pattern undulation of the DM/endothelium. This pattern has been described in relation to corneal graft oedema which is resolved with treatment (Fig. 1).

**Fig. 2:**
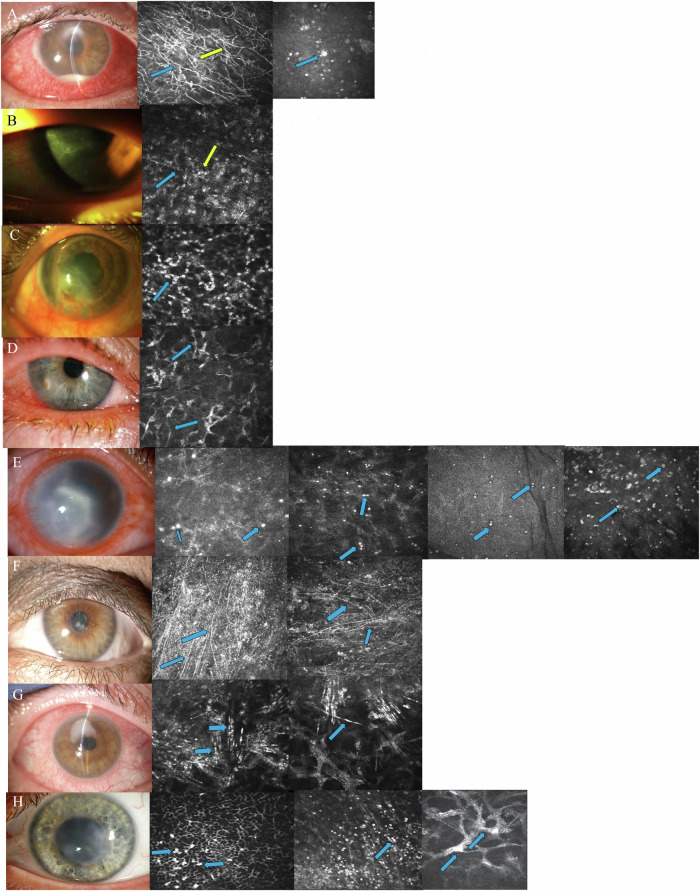
Representative images of the atypical in vivo confocal microscope (IVCM) findings and Representative images of the different cell and inflammatory cells in cases of different pathologies. **A** Slit lamp photo of a confirmed mixed infection (Acanthamoeba Keratitis (AK) and Fungal keratitis (FK). Slit image showing a superior infiltrate and hypopyon. IVCM images of extensive fungal hyphae (Blue arrow) at the superficial layers with visible acanthamoeba cysts (Yellow arrow), and coexisting acanthamoeba cysts in the deeper corneal layers (Blue arrow). **B** Slit lamp photo of another case of AK showing keratoneuritis and perineural infiltrates, corresponding IVCM image showing an aggregation of acanthamoeba cysts (Yellow arrow) around an inflamed nerve. (Blue arrow). **C** Slit lamp images of a case of Candida spp. showing an ulcer at the graft host junction of a partial graft with a dense paracentral infiltrate. IVCM images showing numerous highly reflective round structures at mid corneal stromal level (yeasts) (blue arrow). Slit lamp images of another case of FK (Aspergillus) showing a paracentral small infiltrate and conjunctival injection. IVCM images showing sparse hypereflective segmented lines (fungal filaments) with dichotomous branching. (Blue arrows). **D** Slit lamp images of another case of FK (Aspergillus) showing a paracentral small infiltrate and conjunctival injection. IVCM images showing sparse hypereflective segmented lines (fungal filaments) with dichotomous branching. (Blue arrows). **E** A confirmed case of Acanthamoeba Keratitis (AK), Slit lamp images showing a ring ulcer with a dense infiltrate. The first left IVCM image showing scattered hypereflective acanthamoeba cysts. The middle image of another frame of the same case showing a different presentation of acanthamoeba arrangement in clusters and lines, the right image shows another frame of the deep stroma showing visible scattered hypereflective cyst despite a homogenous hyperreflective extracellular matrix and the final image showing scattered acanthamoeba cysts (White arrows) and visible trophozoites. (Blue arrows). **F** A confirmed case of Fusarium Sp. Fungal keratitis (FK): Slit lamp images showing a central small infiltrate. The left IVCM image showing hypereflective hyphae on extracellular matrix at the superficial corneal stroma and the right IVCM image showing hyperreflective hyphae and inflammatory cells in the deeper stromal layers. (Blue arrows). **G** A confirmed case of fungal Aspergillus sp. keratitis (FK) Slit lamp images showing a para-central superior infiltrate. The left IVCM image showing hypereflective vertical segmented hyphae (showing a train track appearance) within the deep stromal layers indicating Aspergillus sp. and evident cystic spaces (oedema) at the superficial corneal stroma. The right IVCM image showing hyperreflective horizontal hyphae in the deeper stromal layers (irregularly distributed) (Blue arrows). **H** A confirmed case of bacterial keratitis (BK) Pseudomonas species. Slit lamp images showing a para-central infiltrate. The left IVCM image showing hypereflective inflammatory cells within the epithelium. Middle IVCM image showing scattered inflammatory cells at the superficial corneal stroma. Right IVCM image showing activated dendritic cells with hyperreflective processes and cell bodies. (Blue arrows). No organism specific cells are seen.

Acanthamoeba and fungal microbes could only be discerned by IVCM. No specific features related to bacterial keratitis were noted. Activated keratocytes, hyper reflective cell bodies and connecting cell processes were also only evident on IVCM. On ASOCT, hyper reflective areas of stroma indicated keratocyte activity and stromal infiltration. (Fig. 2)

**Fig. 3:**
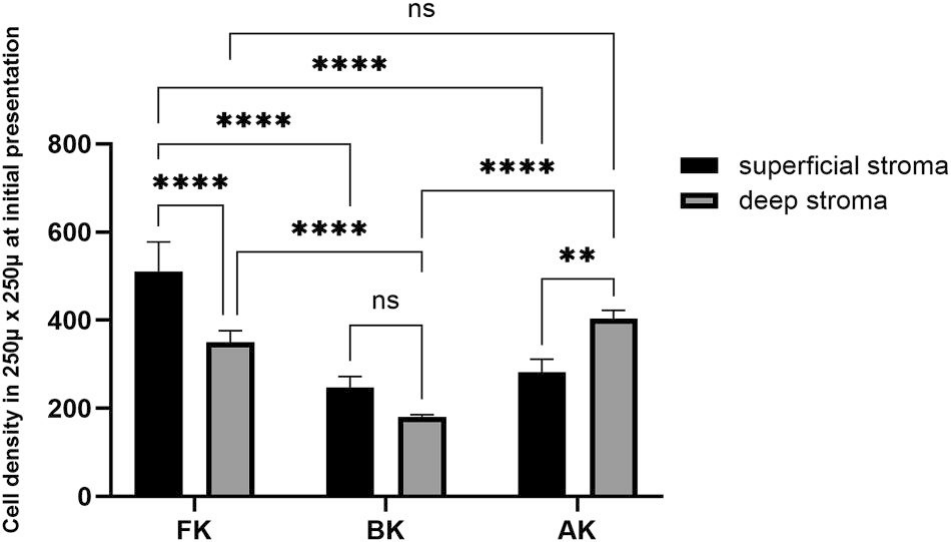
Bar chart showing cell density comparison across organism groups and stromal depth. Mean cell density (cells/mm²) in the superficial stroma (SS) and deep stroma (DS) for cases of fungal keratitis (FK), Acanthamoeba keratitis (AK), and bacterial keratitis (BK) as assessed by in vivo confocal microscopy (IVCM). Data are presented as mean ± standard deviation (SD). Error bars represent standard deviation.

Significant differences in cell density in superficial and deep stroma were noted between AK, FK and BK. However, given the nature of the images we had to include keratocytes in the counts. It is well known that the anterior stroma has a greater density of keratocytes compared to the posterior stroma [8]. This would have skewed the results of the actual value of infiltrating cells but as it was a common factor to all patients and all scans, the relative differences noted are still valid. Interestingly, the anterior stroma showed a reduced cell density in AK compared to the posterior stroma. This is most likely related to the fact that Acanthamoeba feed on the keratocytes [34], which would have reduced the number of keratocytes in the count. (Fig. 3) Mastropasqua et al. have also used IVCM to quantify the cell density in the anterior and posterior stroma in neurotrophic keratitis, to stage the disease [33]. Cell density measurement is an advantage of IVCM over AS-OCT. With the latter, corneal thickness has been used as a surrogate to quantify disease severity and response to treatment [35].

Correlation of microbes identified by PCR or culture with IVCM findings has been attempted for fungal and Acanthamoeba IK in particular. In a large prospective study of 239 patients with severe IK (AK and FK), Chidambaram et al. assessed the sensitivity, specificity, and positive and negative predictive values of IVCM compared with those of a reference standard of positive culture or light microscopy and have concluded that IVCM, when performed with experienced confocal graders has high sensitivity, specificity, and test reproducibility for detecting fungal filaments and Acanthamoeba cysts. Particularly for detecting organisms in deep ulcers in which culture and light microscopy results were negative [28]. Kanavi et al. and Vaddavalli et al. reported similar results emphasising on the diagnostic capabilities of IVCM [12, 14]. Though our study included culture positive cases of BK and two cases of mixed keratitis in addition to FK and AK, the limited number of cases in each group poses a limitation in this study. (Fig. 2)

Our study briefly sheds light on different findings of IK at a single time point, by both IVCM and AS-OCT. Both devices have shown to add value in objectively assessing different parameters of IK caused by some microorganisms, asserting the importance of their use in order to achieve a well rounded clinical practice. However, a larger cohort monitoring the changes in the cornea during the course of healing of IK, from active to healed stage can provide further information on the various parameters examined. This might also enable us to achieve a quantitative analysis through comparing the cell density on IVCM and integrated density using AS-OCT at different stages of IK. Wherein, artificial intelligence (AI) are showing favourable outcomes in a number of eye conditions, building an AI databases using AS-OCT and IVCM, along with microbiology can enhance early diagnosis and better management of IK. AI can potentially be trained to pick up antigen specific features seen with both imaging modalities in order to conclude the nature of infection. Thus, providing more detailed information for a well rounded clinical evaluation. Down the line, the use of AI may help well trained practitioners dodge the pitfalls of culture and PCR.

### What was known before

- IK requires prompt diagnosis, while in vivo confocal microscopy (IVCM) and anterior segment optical coherence tomography (AS-OCT) are two useful imaging modalities in examination of the cornea.

### What this study adds

- While previous research has highlighted the individual benefits of IVCM and AS-OCT, this study addresses the need for a comparative evaluation in severe infectious keratitis.
- It robustly demonstrates IVCM’s distinct superiority in identifying microbial elements and cell density changes, complementing AS-OCT’s strength in assessing tissue architecture and oedema, thus providing clearer guidelines for their combined application.

## Data Availability

The datasets generated during and/or analysed during the current study are available from the corresponding author on reasonable request.
